# The impact of short-term incentives on physical activity in a UK behavioural incentives programme

**DOI:** 10.1038/s41746-019-0164-3

**Published:** 2019-09-16

**Authors:** Cother Hajat, Ali Hasan, Shaun Subel, Adam Noach

**Affiliations:** 1 The Vitality Group, Chicago, IL USA; 2 Vitality Health, London, UK

**Keywords:** Risk factors, Cardiovascular diseases, Epidemiology

## Abstract

This observational study investigates whether the provision of ongoing short-term-incentives for verified physical activity increases and sustains levels of physical activity. We compared UK members at baseline (years 1 and 2) prior to Vitality’s Active Rewards (VAR) intervention commencing (year 3) and follow-up (year 4) for verified, self-reported (encompassing additional physical activities), mortality relative risk and satisfaction with physical activity. Members were categorised into low-active, medium-active and high-active by tertiles of baseline physical activity. Of 11,881 participants, 6477(54.5%) were male, with mean age 39.7(SD 9.8) years. At follow-up, annual active days had increased by 56% overall [60.8(59.7–61.9)–94.8(93.0–96.5)]; 554% in low-active [8.5(8.3–8.7)–47.1(44.7–49.5)]; 205% in medium-active [39.8(39.4–40.2)–81.4(78.7–84.1)] and 17% in high-active members [131.7(129.9–133.5)–153.7(150.7–156.7)] (all *p* < 0.001). Annual weeks of attaining international physical activity recommendations increased by 19% overall [22.2(42.8%)–26.4(50.8%)] and by 316% for low-active members [4.9(9.5%)–15.5(29.8%)]. Self-reported active minutes/week increased by 45% overall [1423(139.4–145.2)–207.0(201.8–212.3)] and 712% in low-active members [20.1(19.3–21.0)–143.2(134.6–151.9)]. Happiness with exercise levels also increased from 1985(49.4%) to 3414(84.9%) members (all *p* < 0.001). The relative risk of mortality from a lack of physical activity reduced by 7% for low-active members [from 0.99 to 0.92], 5% for medium-active [0.94–0.89] and 3% for high-active [0.89–0.86](*p* < 0.001) and by 0.02% for each additional year of age (*p* = 0.02). This large-scale, real-world, short-term-incentives intervention led to a dramatic increase in physical activity which was sustained for, and still increasing after, two years. If applied at broader level, this approach could considerably aid progress towards WHO targets in its Global Action Plan for Physical Activity.

## Introduction

The use of incentives for behaviour change is becoming an increasingly employed tool to improve health outcomes, often using technology such as wearables and smartphones.^1^ Whilst the impact from behaviour change incentives has been discussed in the literature, the extent to which the impact was attributable to the incentive, technology and population is unclear in experimental settings. Moreover, behavioural interventions ideally need to be studied in real life scenarios rather than in research settings, which can be both challenging and costly to undertake. Nonetheless, the body of evidence does lean towards positive effects from the use of incentives and wearables on lifestyle behaviour change and health outcomes.^2^

Vitality is a UK-based, wellness-focused insurer that offers incentives for undertaking verified physical activity, healthy food purchases, health screening and other health-promoting behaviours.

Vitality’s long-term-incentives programme is based on behavioural economics science and offers discounts on gym membership, flights, hotel stays and more. These incentives are administered on attainment of annual status based on points earned from health-promoting behaviour, as well as regular attainment of points to demonstrate activity levels on a weekly basis. The impact of engagement with the standard long-term-incentives programme has previously been reported including healthier food purchases, reduced healthcare costs, hospital admissions and costs associated with hospital stays.^3,4^ In 2015, the Vitality Active Rewards (VAR) programme was launched which offers, in addition to the standard long-term offering, short-term-incentives of cinema tickets and Starbucks hot drinks for the attainment of weekly verified physical activity goals.

The objective of the current study was to evaluate whether the use of ongoing, short-term incentives had a positive and sustained impact on physical activity levels in Vitality members.

## Results

A total of 11,881 participants were included, of whom 6477 (54.5%) were male and mean age was 39.7 (SD 9.8) years (Table 1).

Mean verified active days of physical activity annually were 9, 40 and 132 for the low, medium and high physical activity groups, respectively. Self-reported mean minutes of weekly physical activity were 20, 110 and 291, respectively, for the same baseline groups.

**Fig. 1:**
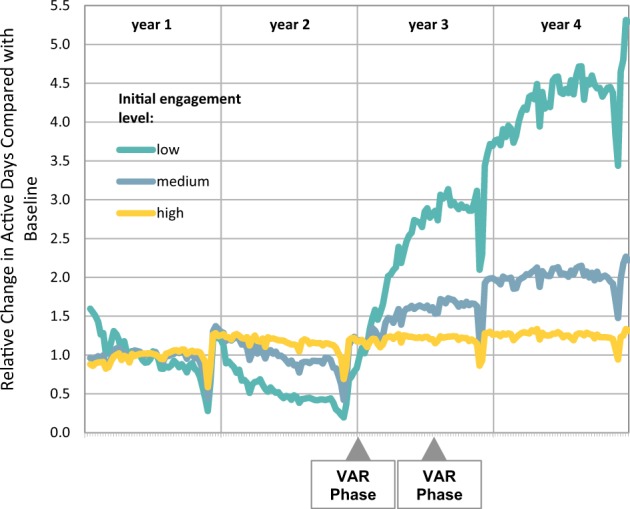
Relative change in Active Days before and after VAR activation by baseline activity level. *Note*: Data were analysed using verified active days on a daily basis; significant dates include VAR Phase 1: activation of VAR Starbucks hot drinks reward, Phase 2: activation of cinema tickets; Dips in activity occur over the December holiday period. A comparison between the groups for relative change between baseline and year 4 was significant at *p* < 0.001

Figure 1 shows that at follow-up compared with baseline, annual active days had increased by 56% overall [from 60.8 (95% CI 59.7–61.9) to 94.8 (95% CI 93.0–96.5)]; 554% in low-active [from 8.5 (95% CI 8.3–8.7) to 47.1 (95% CI 44.7–49.5)]; 205% in medium-active [from 39.8 (95% CI 39.4–40.2) to 81.4 (95% CI 78.7–84.1)] and 17% in high-active members [from 131.7 (95% CI 129.9–133.5) to 153.7(95% CI 150.7–156.7)] (all comparisons were significant at *p* < 0.001).

**Table 1:** Baseline characteristics

Characteristic	
*N*	11,881
Male *n* (%)	6477 (54.5%)
Age mean (SD) years	39.7 (9.8)
Age groups	
<25	361 (3.04%)
25–34.9	4011 (33.76%)
35–44.9	4216 (35.49%)
45–54.9	2383 (20.06%)
55–64.9	742 (6.25%)
65 or above	168 (1.41%)
Baseline total (days with points earning events per year)^a^	*N* (%); mean (SD)
Low	3893 (32.77%); 8.50 (5.78)
Medium	3946 (33.22%); 39.84 (12.87)
High	4041 (34.02%); 131.69 (57.34)
Overall	11,880 (100%); 60.81 (62.74)
Baseline minutes per week^b^	*n* (%); mean (SD)
Low (≤67.5 min)	3200 (32.84%); 20.12281 (23.90)
Moderate (67.5–160 min)	3218 (33.02%); 110.4809 (26.65)
High (160+ minutes)	3327 (34.14%); 290.5311 (152.50)
Overall	9745 (100%); 142.2799 (145.25)

^a^Tertiles of average verified active days in a 12-month period (years 1 and 2)

^b^Tertiles of self-reported minutes per session multiplied by sessions per week (years 1 and 2)

The relative increase was higher in females at 57% [from 57.0 (95% CI 55.4–58.7) to 89.6 (95% CI 87.0–92.3) than males at 55% [from 64.0 (95% CI 62.4–65.5) to 99.0 (95% CI 96.6–101.4)] (*p* < 0.001); and increased significantly for all age groups (*p* < 0.001) with the exception of the over 65 group [*n* = 168; *p* = 0.8].

### Types of physical activity

Table 2 shows the types of activity that contributed to the increase in active days at follow-up. Steps and tracker points accounted for the greatest increases at 307% [from 19.3 (95% CI 18.3–20.3) to 59.3 (95% CI 57.7–60.9)] and 290% [from 3.8 (95% CI 3.5–4.1) to 11.0 (95% CI 10.4–11.6)], respectively. Points accrued for parkruns and gym use changed to a lesser extent.

**Table 2:** Breakdown by types of physical activity contributing to active days pre-VAR and post-VAR

	Gym	Steps	Track	Park
	Mean (SD) (95% CI)	Mean (SD) (95% CI)	Mean (SD) (95% CI)	Mean (SD) (95% CI)
Pre-VAR	38.6 (46.3)	19.3 (53.9)	3.8 (18.6)	0.3 (2.5)
	(37.8–39.4)	(18.3–20.3)	(3.5–4.1)	(0.3–0.4)
Post-VAR	29.7 (48.4)	59.3 (90.3)	11.0 (32.7)	0.5 (3.3)
	(28.9–30.6)	(57.7–60.9)	(10.4–11.6)	(0.4–0.5)

Data on verified active days were used, broken down by type of physical activity reported. All comparisons were significant at *p* < 0.001

### Self-reported physical activity

Self-reported active minutes per week increased by 45% overall [from 1423 (95% CI 139.4–145.2) to 207.0 (95% CI 201.8–212.3)] and 712% in low-active members [from 20.1 (95% CI 19.3–21.0) to 143.2 (95% CI 134.6–151.9)] (Table 3).

**Table 3:** Self-reported minutes per week of physical activity

	Low	Medium	High	Total
	Mean (SD) (95% CI)	Mean (SD) (95% CI)	Mean (SD) (95% CI)	Mean (SD) (95% CI)
Pre-VAR	20.1 (23.9)	110.5 (26.7)	290.5 (152.5)	142.3 (145.3)
	(19.3–21.0)	(109.6–111.4)	(285.3–295.7)	(139.4–145.2)
Post-VAR	143.2 (250.0)	166.1 (200.5)	308.0 (304.0)	207.0 (265.9)
	(134.6–151.9)	(159.2–173.1)	(297.6–318.3)	(201.8–212.3)

Self-reported moderate or vigorous intensity activity data were included; all comparisons were significant at *p* < 0.001

### Change in relative risk for mortality

**Fig. 2:**
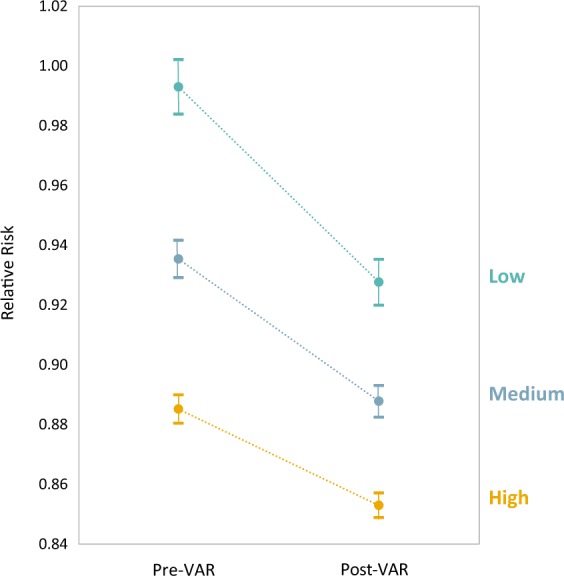
Impact of VAR on relative risk of mortality due to lack of physical activity Verified data were used to derive the relative risk of mortality attributable to physical activity. The theoretical range of relative risk from physical activity is 0.77 to 1.26.^7–15^ All comparisons were significant at *p* < 0.001

Figure 2 shows the reduction following VAR in relative risk for mortality due to a lack of physical activity (PARR), which reduced by 7% for low-active [from 0.99 (95% CI 0.98–1.00) to 0.92 (0.92–0.94)], 5% for medium-active [from 0.94 (95% CI 0.93–0.94) to 0.89 (95% CI 0.88–0.89) and 3% for high-active members [from 0.89 (95% CI 0.88–0.89) to 0.86 (95% CI 0.85–0.86)] (*p* < 0.001).

The shift in PARR was greater with each additional year of age (0.02%; sex-adjusted *p* = 0.02). A non-significant greater shift was observed in males compared with females aged over 45 years (0.4%; age-adjusted *p* = 0.8).

### Meeting international recommendations for physical activity

Table 4 shows the number of weeks annually that members achieved WHO physical activity recommendations; at follow-up these had increased by 19% overall from 22.2 (42.8%) to 26.4 (50.8%) weeks and improved the most for low-active members by 316% from 4.9 (9.5%) to 15.5 (29.8%) weeks (*p* < 0.001). In high-active members there was a small but significant decrease of 2.7% from 40.4 (77.7%) to 39.3 (75.6%) weeks.

**Table 4:** Verified Weeks of meeting physical activity recommendations

	Low	Medium	High	Total
	*n* (%)	*n* (%)	*n* (%)	*n* (%)
Pre-VAR Weeks mean(SD)	4.9 (3.4)	20.3 (8.3)	40.4 (9.1)	22.2 (16.3)
Post-VAR Weeks mean(SD)	15.5 (16.8)	23.8 (16.4)	39.3 (14.1)	26.4 (18.6)

WHO recommendations of 150 min of moderate or 75 min of vigorous physical activity per week.^16^ Verified data on active days were used. All comparisons were statistically significant at *p* < 0.001

### Satisfaction with physical activity levels

At baseline, members’ satisfaction with current levels of exercise included 865 (21.5%) stating they needed improvement but were not doing anything to change, 1172 (29.1%) looking to improve but needing help and 1985 (49.4%) stating that they were happy with exercise levels. Following engagement with Active Rewards, members’ satisfaction levels had shifted significantly to zero members stating that they need improvement but were not doing anything to change, 608 (15.1%) looking to improve but needing help and 3414 (84.9%) stating that they were happy with exercise levels (*p* < 0.001).

## Discussion

This large, real-world study of ongoing short-term incentives to promote physical activity demonstrates novel and important findings. Firstly, short-term incentives both increased and prolonged members’ engagement in physical activity over and above the use of trackers and long-term incentives. Secondly, the order of magnitude of the change was considerable enough to have a likely impact on health outcomes.

Engagement with VAR resulted in a dramatic increase in active days for all baseline activity levels, in particular those who were low or moderately active at the outset. The increase in physical activity was sustained and continued to increase throughout the 2-year period after the introduction of VAR. Furthermore, the increase in physical activity correlated chronologically with the introduction of the two phases of VAR, suggesting that staggering incentives may play a role in sustaining their impact. The projected health outcome of relative risk of mortality attributable to a lack of physical activity was significantly reduced in all members, regardless of baseline activity level. VAR engagement also increased the likelihood of meeting the WHO weekly physical activity recommendations particularly in those who were initially low or moderately active.

As members age, levels of physical activity would be expected to decline, the prevention of which would also be considered a positive outcome. Our results found less impact from VAR in high-active members and those aged over 65 years, although they were still more physically active at 76% than nationally published rates of physically active UK population at 63%, which are self-reported hence may be over-estimates.^5^

Most previous studies have been unable to report on sustainability of impact due to less than 12 months of follow up. Our study shows that impact was sustained for at least 2 years although the staggering of the introduction of VAR in phases could have contributed to some of the sustained impact. Furthermore, the near-doubling of satisfaction with activity level post-VAR supports likely sustainability beyond the follow-up period, which has also been suggested in one review.^6^

Previous research on members from the US Vitality programme showed that physical activity acts as a trigger for other healthy behaviour, such as increased fruit and vegetable intake and health screening.^7^ Further research on whether this transpired after engagement with VAR would be of interest.

Future follow-up data for the same members will be able to verify whether the predictions in improved health do indeed manifest. Further thought is also required as to how this type of ongoing, near-time feedback may play a role in the existing arsenal of feedback that is currently available and utilised to maintain motivation for physical activity.

Several studies in the economic literature have considered the impact of incentives in improving exercise. One such study highlighted the clear promise of impact of financial incentives in short-term scenarios but cautioned of the short-term persistence of incentivised exercise behaviours following the withdrawal of an incentive.^8^ Other work considered the effectiveness and persistence of financial incentives in the absence of contingent contracts, suggesting that single, one-off interventions may be less effective than sustained ones.^9^ A number of studies have failed to demonstrate persistence of weight loss following such interventions.^10^ The Illinois Workplace Study, which reviewed an incentive-based workplace programme on over 12,000 employees found that only two out of 39 outcomes were improved: the number of employees receiving health screening, and a perception of management prioritising health and safety.^11^ These studies and others, including a systematic review^12,13^ have demonstrated the promise of significant impact of incentives but have not generally provided clarity on the interventions needed, precision on their impact or data on long-term outcomes consistent with a theory of sustained behaviour change through the use of incentives.

Wearables have become increasingly popular over the previous decade. However, few studies have investigated the impact of both wearables and short-term incentives simultaneously. One study of 800 people testing wearables only, charity donation incentives, cash incentives and no intervention (controls) reported after 6 months that changes in physical activity were only maintained in those receiving the monetary incentives.^14^ Another trial on the impact of small incentives related to pedometer activity demonstrated that persistent increases in activity can be seen post-intervention.^6^

Previous studies on wearables alone reported conflicting results on health impact.^2^ A large review including over 200 studies found that internet interventions improved diet, physical activity, adiposity, tobacco use, and excess alcohol; and mobile interventions improved physical activity and adiposity.^15^ A review of the use of wearable technology on health outcomes and a small randomised controlled trial on wearables showed some improvement in physical activity but not on body mass index (BMI) or other health outcomes.^14,16^ A meta-analysis on the use of health technology for remote monitoring found no improvement in the management of the underlying health conditions.^17^

Reasons for the contradictory findings in individual studies include the use of conventional research study settings and designs, which are better suited for more conventional treatments such as medication, than for real-life interventions administered to populations rather than patients. Furthermore, the use of technology by itself is far less impactful and meaningful in real-world settings than the use of broader packages of interventions in which technology plays a part but is not the only offering. The vast majority of published studies are also small, generally under 1000 subjects, and investigate hard outcomes, such as BMI, which are not expected to change in the short term.

The current study is to our knowledge the largest study to investigate the impact of short-term incentives on physical activity. Furthermore, the study is based in a real-world rather than a research setting, rendering the findings highly generalisable and replicable. Also, the incentives are real rather than theoretical, further strengthening the study.

The findings of the current study may help to contextualise some of the challenges seen in the literature around understanding the impact of wearables and activity tracking independently. The findings support the possibility of the impact of wearables and short-term incentives being synergistic.

Previous studies by Vitality and others have shown behavioural incentives can significantly improve health-promoting behaviour, such as fruit and vegetable intake, health screening and other wellness activities,^3,4,7^ as well as longer term outcomes of hospital admission and lengths of hospital stay.^3,4^

The current study shows that ongoing, small, short-term incentives are more impactful than the conventionally employed longer-term incentives. Members who were least physically active at baseline saw the greatest improvements in all measures and are most likely to benefit from such initiatives.

Despite the WHO setting targets in its Global Action Plan on Physical Activity to reduce physical inactivity by 10% by 2025 and 15% by 2030,^18^ physical inactivity is increasing. A recent review, pooling results from 358 studies, reported that in high-income countries, such as the UK, 37% of the population remain physically inactive and this worsened between 2001 and 2016.^19^ Novel approaches to tackling physical inactivity are required.

The results of the current study suggest that if deployed at larger scale, such short-term incentives could provide a tremendous shift in the physical activity levels of the population required to meet such targets. It is particularly encouraging that the least active members were motivated the most, suggesting that such an initiative could have great impact on a large proportion of the population.

There are several strengths and limitations for the current study. This is one of the largest and most comprehensive real-world studies of the impact of ongoing short-term incentives on physical activity and is more generalisable than studies conducted in pure research settings. The long-term nature of the study demonstrates that, at a population level, improved physical activity can be sustained. The extrapolation of activity levels to mortality endpoints is novel and adds to the potential quantification of these benefits.

Another major criticism of previous studies is that physical activity levels are self-reported. The current study is novel in that it relies on verified data but has also shown proportionate improvements in self-reported physical activity levels in the same participants.

Verified points for physical activity were monitored using synchronised activity trackers, verified gym attendance or parkruns. However, this may underestimate physical activity as some users may not be recording any or all of their physical activity. Other members may be participating in physical activity that is not recorded or rewarded, such as strength and flexibility-based exercises and/or attendance at non-partner fitness facilities. We accounted for these in the study design by using both verified physical activity measures as well as self-reported ones. Furthermore, we were able to do so by reporting on the same members’ behaviour before and after the introduction of VAR.

A limitation is that the relative intensity of activity for those engaging with exercise of moderate or high intensity cannot be precisely discriminated between individuals. The advent of more advanced technology that monitors heart rate and heart rate variability may be of future utility in this context.

As noted in other literature, short-term incentives may not produce long-term behaviour change, and the removal of incentives may reduce or eradicate the intended behaviour change impact of those incentives. Vitality’s wellness programme is integral to Vitality products and while incentives provided may be modified, the incentives programme itself is a core product and will remain ongoing.

Furthermore, this study provides insights into the satisfaction of participants with their physical activity levels, which is key to ensuring sustained impact beyond the period of study.

Whilst the VAR programme is offered to all Vitality members, not everyone chooses to take up the offering. As such the results may not be representative of all Vitality members or the general population. Understanding the key factors that encourage members to engage with the VAR programme is a key area for future investigation.

In summary, this large real-world intervention demonstrated that the use of ongoing short-term incentives led to a dramatic improvement in physical activity, particularly in those with most to gain, the least physically active. The impact was sustained and continued to increase 2 years following introduction of the intervention. Those who were least active increased their weeks of meeting WHO physical activity recommendations by more than three-fold. Few other examples of real-world interventions for physical activity have shown this magnitude of change over a sustained period of time.

If applied at broader level, initiatives using the same intervention could significantly aid progress towards physical activity targets set for all countries by the WHO in its Global Action Plan for Physical Activity.^19^ Further work is required to identify the triggers that encourage people to participate in incentives-based programmes, with a view to being able to offer similar initiatives at population level.

## Methods

### The vitality programme

This was a prospective longitudinal study of members enroled in UK Vitality’s short-term-incentives programme, VAR.

Points for verified physical activity are accrued by

- Verified visits to an affiliated gym (membership card swipe)
- Verified wearable tracker activity (including Fitbit, Garmin, Polar, Apple watch and others), such as 7000, 10,000 or 12,500 daily steps or other measures of intensity of physical activity such as heart rate.
- Verified Parkrun activity (a social running programme)^20^ or other organised endurance events.

VAR incentives are automatically administered in real-time via a dedicated mobile application or via the Vitality website member-zone.

Details for the equivalent VAR programme in South Africa are previously published.^21^

### Research outcomes

Primary Research outcomes were two-fold: whether VAR increases verified physical activity levels and, if so, what is the duration of impact?

Secondary Research outcomes were whether VAR improves predicted health outcomes and satisfaction with exercise levels.

The effect of VAR was compared against the standard Vitality long-term incentives offering using a before and after VAR comparison on the same members.

**Fig. 3:**
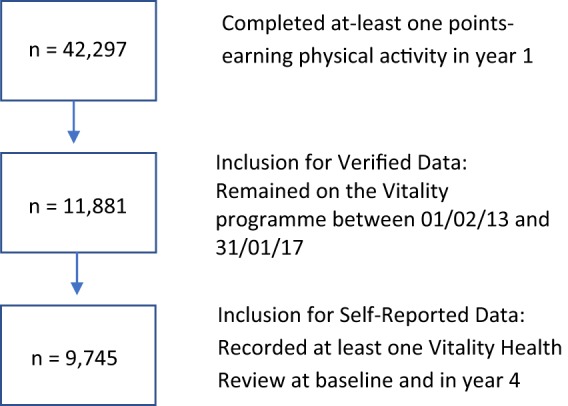
Inclusion criteria

Inclusion criteria required members to (Fig. 3):

- Have been actively engaged with the standard Vitality offering in year 1 by participating in at-least one points-earning physical activity.
- For verified data: Have been on the Vitality programme for the full 4-year study period from 1 February 2013 to 31 January 2017, which represents 24 months pre- and post-introduction of VAR.
- For self-reported data: Have completed at least one Vitality Health Review at baseline and at year 4.

### Types of data

Verified data included:

- Number of days in which physical activity points were accrued. The use of active days rather than points accounts for the variance in number of points accrued for different and duplicate activities.
- Points accrued for gym, steps, park run^20^ and other tracker activity.
- Impact of physical activity levels on physical activity relative risk (PARR) for mortality, aggregated from published impact of lowering of mortality risk with increased physical activity.^22–30^

Members also received points for completing regular Health Reviews during which information on self-reported physical activity is recorded. Points are only received for completion of the Health Review questions and not on the responses provided, e.g. self-reported minutes of PA.

Self-reported Health Review data included:

- Minutes and intensity of physical activity per week.
- Satisfaction with exercise levels.

Self-reported responses were taken from Health Review questions as the closest reading at the beginning of baseline and end of the 24-month post-VAR period (allowing 3 months inside, and 6 months outside, of the study period). The Health Review questions (and responses) used for these analyses included:

- How many days a week do you exercise (0–7).
- How long are your exercise sessions? (minutes).
- How intense are your exercise sessions? (light e.g. regular walking; moderate e.g. brisk walking, cycling for transport; vigorous e.g. running, fast swimming, fast cycling).
- Are you happy with your level of exercise? (Happy; Needs improvement but not changing; Want to improve but need help).

We studied self-reported PA for the same members before and after the introduction of VAR for two reasons. Firstly, we wanted to deal with the potential bias of members being more likely to report existing PA because of the incentives, rather than increasing the levels of PA. As self-reported PA is not incentivised, there would be no reason for members to increase reporting outside of increased PA. Furthermore, we wanted to account for types of PA that are not recorded or rewarded, such as strength and flexibility-based exercises and/or attendance at non-partner fitness facilities. However, self-reported PA data were not used for the main primary or secondary research outcomes and are only reported in Table 3.

A before-and-after VAR comparison was also made for the verified proportion of weeks annually that members were achieving World Health Organisation physical activity recommended levels (150 min moderate or 75 min vigorous) physical activity,^19^ making the assumption that earning verified points on a day corresponds to at least 30 min of moderate-intensity or 15 min of vigorous-intensity activity; points needed to have been earned on 5 days per week to meet recommended levels.

In order to provide a single composite score of health, a proprietary Vitality Age Score is utilised of which physical activity is one component.^30^ The relative risk of mortality from the physical activity component was derived as the contribution of verified physical activity to the total mortality from all causes combined.^22–30^

All Vitality members are consented at enrolment into the Vitality Programme for use of their data for research purposes in anonymized format, and in compliance with ethical regulations.

### Statistical analyses

Analyses were conducted in PL/SQL (Developer Version 7.1.5.1398) and STATA (version 14).

The analysis accounts for the variance and duplication in points-earning by using days of earning points for activity rather than total number of points.

Members were assigned to a priori activity level categories according to baseline (years 1 and 2) tertiles of verified active days and self-reported minutes per week of physical activity. Verified active days tertiles were low-active (0–16.9), medium-active (17–61.9) and high-active (62+); self-reported minutes per week (of moderate and high intensity activity) tertiles were low (≤120), medium (120–240) and high (240+). Baseline activity was compared with the post-VAR period of year 4 only to account for the staggered introduction of VAR during year 3. Unless stated otherwise, further analyses were stratified by baseline-verified active days.

Comparisons for categorical data were conducted using logistic regression, and for continuous data using linear regression, adjusting for age and sex. Data on socio-economic status were not available but unlikely to vary by a large extent as these are privately insured members.

The change in relative risk for physical activity (PARR) was reported as a ratio of post-VAR PARR: pre-VAR PARR, segmented by baseline physical activity level.

### Reporting summary

Further information on research design is available in the Nature Research Reporting Summary linked to this article.

## Supplementary information

Reporting Summary

## Data Availability

The data that support the findings of this study are available from Vitality Health, UK but restrictions apply to the availability of these data, which were used under license for the current study, and so are not publicly available. Data are however available from the authors upon reasonable request and with permission of Vitality Health, UK.

## Supplementary information

**Supplementary information** accompanies the paper on the *npj Digital Medicine* website (10.1038/s41746-019-0164-3).
